# Internship during Certificate Course in Medical Editing by UHS and PAME

**DOI:** 10.12669/pjms.40.11.11266

**Published:** 2024-12

**Authors:** Nabiha Farasat

**Affiliations:** Prof. Nabiha Farasat CMJE, CME, MHPE, M. Phil, BDS. Director Research & Head Oral Pathology Department, Bolan Medical College, Quetta, Pakistan. Email: nabihasaeed40@gmail.com

Pakistan Association of Medical Editors (PAME) in collaboration with University of Health Sciences Lahore initiated a Certificate Course in Medical Editing in 2019 and so far six batches have qualified. Later on it started an advance course as well named as Certificate in Medical Journalism for Editors (CMJE).^1^ The second four days contact session of this CMJE course includes spending one day each at the three leading Impact Factor Journals in Karachi i.e. Pakistan Journal of Medical Sciences, Journal of Pakistan Medical Association and Journal of College of Physicians & Surgeons Pakistan. CPSP is an important partner in this training course.

Ours was the first batch of the *internship* program from June 15^th^ to June 18^th^ 2022.^2^ The purpose is to create a step-by-step approach towards Master’s Degree program. The objectives of this internship were to study the working of these impact factor journals in Pakistan which all follow different business models, acquaint with their management and publication process, and develop professional links. Twelve (12) participants (details mentioned in Table-I) visited and spent a day each at the Journal of College of Physicians and Surgeons Pakistan (JCPSP), Journal of Pakistan Medical Association (JPMA), and Pakistan Journal of Medical Sciences (PJMS) where they were briefed about their functioning by the respective journal editorial staff. Names of participants in this internship programme are presented in Table-I while their affiliation and province to which they belonged are covered in Table-II.

**Table-I:** List of Participants in the Internship programme

Sr. No:	Name	City
1.	Dr. Abubakar Ali Saad	DG Khan
2.	Dr. Afshan Shahid	Lahore
3.	Prof. Mariyah Hidayat	Lahore
4.	Dr. Memoona Mansoor	Islamabad
5.	Dr. Mukhtar Mehboob	Quetta
6.	Dr. Nabiha Farasat	Quetta
7.	Prof. Nadia Naseem	Lahore
8.	Dr. Neelofar Yousaf	Lahore
9.	Dr. Noor-i-Kiran	Faisalabad
10.	Dr. Rehana Khadim	Rawalpindi
11.	Dr. Wajid Jawaid	Karachi
12.	Dr. Ghulam Mustafa	Rahim Yar Khan

**Table-II:** Participants and their affiliation.

Province	Total Participants	Institutes
Punjab	6	1. Professor, Editor-in-Chief Biomedica, UHS
		2. Professor, Associate Editor at ESCULAPIO, official journal of Services Institute Medical Sciences, Lahore
		3.Associate Professor, Joint Editor at Shaikh Zayed Medical College, Rahim Yar Khan
		4. Professor, Editor Journal of UCMD at the University of Lahore
		5. Professor of Cardiology at DG Khan Medical College.
		6. ABWA Medical College, Faisalabad
Sindh	1	1. Dow Medical College, Karachi
Islamabad	2	1. Managing Editor, Pakistan Armed Forces Medical Journal, Rawalpindi.
		2. Islamabad Medical and Dental College, Islamabad
Balochistan	2	1. Professor at Bolan Medical College, Quetta
		2. Benazir Hospital, Quetta

After visiting these journal offices, detailed briefing and discussions, the participants prepared a detailed report based on their observations highlighting the positive aspects besides giving suggestions for improvement if any. They also highlighted how they are going to implement in their respective journals what they have learnt during this internship. Group leaders made their presentations at the joint session held at the CPSP Campus on June 18^th^ 2024 which were later supplemented by input from other group members. Editors of all the above mentioned IF Journals and some of their editorial staff were also present on this occasion. It provided an excellent opportunity to the participants to learn how to run and manage a successful journal and also ensure its sustained publication in the long run. A summary of participants’ observations about these journals is presented in Table-III. Prof. Irshad Waheed Secretary College of Physicians & Surgeons Pakistan was the chief guest in the concluding session and joined the candidates in the Group Photograph.

**Figure:**
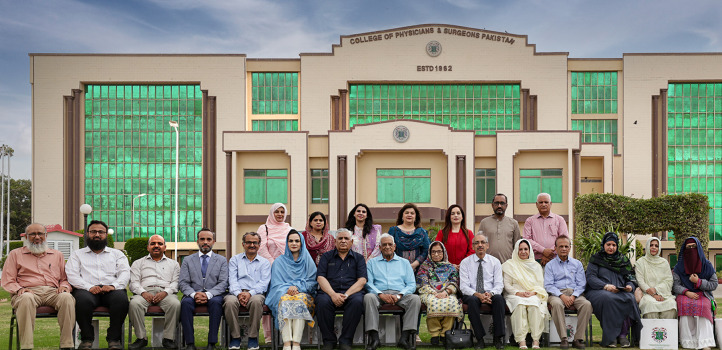
Group photograph with Prof. Irshad Waheed Secretory CPSP, Karachi (18.6.22).

**Table-III:** The comparison of three Impact factor journal.

Journals Qualities	JPMA	PJMS	JCPSP
Established	1953	1984	1992
Impact Factor,	1.002	1.2	1.024
Category	X	W	X
H-Index	48	52	49
Acceptance &	31%	15%	35%
Rejection Rate^1^	69%	85%	65%
ICMJE Criteria^3^	Yes	Yes	Yes
Peer review process	Double-blind	Open Peer Review system	Double-blind
Research publications	All types of studies	All studies except KAP Surveys, animal studies	Except KAP, Narrative and Survey all other
Authorship rule	Scientist, 6 or more	Only 4 authors allowed in most cases	MBBS, BDS up to 6
Waiver Policy	50% discount to student, reviewer on request	Reviewers get discount on request	No
Privileges of Reviewers	Certificates, training	Discount to Reviewers in publication charges, for them processing fee is waived of, Books, Certificates, cash, Registration for training, courses, conferences, workshops	Certificates, payment
Withdrawal Policy	50% payment after peer review	After peer review BLACK LIST	At any stage without any action
Financial resources	33000 processing + publication fee	Rs. 5,000 processing + Rs. 30,000 publication charges	20000 processing + publication fee

## CONCLUSION

Dedication, sincerity, and collaborative work are the keys to the success of JCPSP, JPMA, and PJMS. PJMS stands first in its administrative work, incentives/waivers policy, workshops conduction, and conferences for the medical fraternity. JCPSP has a lenient authors withdrawal policy, an internal review policy, and best authorship rules. Commendable policies of JPMA include student corners, publications of KAP studies, Narrative Review, Experimental Animal studies, and Lab Diagnostics. It is very gratifying that PJMS and JPMA promote journalism by providing training to students and healthcare professionals.
